# Mechanisms of synovial macrophage polarization in osteoarthritis pathogenesis and their therapeutic implications

**DOI:** 10.3389/fimmu.2025.1637731

**Published:** 2025-11-25

**Authors:** Kun Zhang, Zheng Wang, Jiaqi He, Liuru Lu, Wenshu Wang, Aiwei Yang, Huayi Xie, Linhui Huang, Yuying Huang, Ke Zhang, Mingyang Jiang, Ruqiong Wei

**Affiliations:** 1 Department of Trauma Orthopedics, Shenzhen Longhua District People’s Hospital, Shenzhen, China; 2 The Second Clinical Medical College of Guangxi Medical University, Nanning, China; 3 The First Clinical Medical College of Guangxi Medical University, Nanning, China; 4 Department of Bone and Joint Surgery, The First Affiliated Hospital of Guangxi Medical University, Nanning, China; 5 Department of Rehabilitation Medicine, The First Affiliated Hospital of Guangxi Medical University, Nanning, China

**Keywords:** osteoarthritis, synovitis, macrophages, cartilage degeneration, macrophage polarization, immunomodulation

## Abstract

Osteoarthritis (OA) is a degenerative joint disease characterized by synovial inflammation, cartilage degradation, and subchondral bone remodeling. Synovial macrophages, particularly their polarization into pro-inflammatory M1 or anti-inflammatory M2 phenotypes, play a pivotal role in OA pathogenesis. M1 macrophages drive synovitis, oxidative stress, and cartilage catabolism by secreting cytokines (IL-1β, TNF-α) and matrix-degrading enzymes (MMPs, ADAMTS-5), while M2 macrophages promote tissue repair via TGF-β and IL-10. Emerging therapeutic strategies, such as macrophage depletion, mTOR/SIRT1 modulation, and M2 polarization, demonstrate potential in rebalancing the M1/M2 ratio to attenuate OA progression. However, translating these macrophage-targeted strategies into clinical practice remains challenging due to difficulties in achieving subtype-specific targeting, avoiding off-target immune effects, and ensuring consistent therapeutic efficacy across patient populations. However, challenges remain in achieving subtype-specific targeting and translating preclinical findings to clinical applications. This review summarizes current knowledge and provides valuable insights for advancing OA management strategies, underscoring macrophages as promising therapeutic targets in osteoarthritis.

## 1 Introduction

Osteoarthritis (OA), recognized as the most common degenerative joint disease, is characterized by complex pathological alterations involving multiple joint structures including articular cartilage, subchondral bone, synovial membrane, ligaments, and periarticular musculature (1–3). A critical pathological hallmark of OA is synovial inflammation (4–6), with accumulating evidence indicating that the severity of synovitis closely parallels disease progression (7–9). Synovitis, defined as inflammatory changes in the synovial membrane, manifests histologically through distinct features including synovial cell hyperplasia, angiogenesis, and infiltration of inflammatory cells (10–12).

Macrophages serve as versatile immune regulators that participate in both innate immune responses and tissue homeostasis, playing pivotal roles in host defense mechanisms and the maintenance of physiological balance (13). Within this inflammatory microenvironment, macrophages constitute the predominant immune cell population. These cells actively participate in OA pathogenesis through the sustained secretion of pro-inflammatory cytokines and matrix-degrading enzymes, thereby driving disease exacerbation and promoting joint tissue destruction (14, 15). Therefore, an in-depth understanding of the role of synovial macrophages in the initiation and progression of OA is of critical importance. Importantly, among the various immune cells implicated in OA, synovial macrophages represent a particularly attractive therapeutic target due to their numerical dominance, plastic phenotypic switching capacity, and ability to orchestrate both destructive and reparative processes in the joint microenvironment (16). Unlike lymphocytes or neutrophils, which often act as downstream effectors, macrophages act as upstream modulators of inflammation, matrix degradation, and chondrocyte fate. Their dual polarization into pro-inflammatory (M1) and anti-inflammatory (M2) phenotypes provides a therapeutically exploitable axis to restore immune balance and promote tissue repair (17, 18). As such, targeting macrophage polarization offers a more direct and dynamic strategy for modulating joint inflammation and structural damage compared to other immune pathways.

## 2 Composition, functional roles, and phenotypic characteristics of synovial macrophages

### 2.1 Heterogeneity and functional significance of synovial macrophages

The pivotal role of macrophages in OA synovitis has been firmly established since early studies (19, 20). Athanasou et al (21). first identified macrophage markers (CD11b, CD14, CD16, CD68) in OA synovium, though these did not define activation states. Subsequent work by Haywood et al (22)., Benito et al (23)., and Bondeson et al (24). demonstrated markedly increased macrophage infiltration compared with healthy synovium, a finding reproduced in multiple OA animal models (25, 26). Manferdini et al (27). further revealed heterogeneous macrophage subsets (CD14, CD16, CD68, CD80, CD163), emphasizing phenotypic diversity. Polarization profiling has yielded variable results: Van den Bosch et al (28). reported simultaneous elevation of M1 (CD86, CCL3, CCL5) and M2 (CD206, IL−10, IL−1Ra) markers in end−stage OA, whereas Zhang et al (26). observed a pronounced M1 (iNOS ^+^) predominance and M2 (CD206 ^+^) reduction in human and murine OA models. ScRNA‐Seq Revealed the macrophages could be further categorized into two subclusters: C0 and C1. The relative proportion of C0 and C1 was dramatically elevated in the synovium of patients with diabetic OA compared to those with normal synovium (29).

Recent phenotyping advances identified folate receptor−β (FR−β) as a marker of pro−inflammatory monocytes (CD14^high^ CD16^−^) (30). FOLR2 ^+^ tissue−resident macrophages (TRMs) localize perivascularly and interact with CD8 ^+^ T cells (31). This finding enabled non−invasive visualization of macrophage activation via ¹¹¹InCl _3_−DTPA−folate SPECT imaging in rat OA models (32), revealing transient activation peaks in mono−iodoacetate−induced OA and sustained activity up to 12 weeks post−ACL transection. De Visser et al (32). further reported a 28.4% rise in FR−β ^+^ macrophages under high−fat diet conditions in a groove model, concentrated within synovial and subchondral bone regions. Clinical investigations have strengthened the association between macrophage activation and OA progression. Daghestani et al. (33) established significant correlations between synovial fluid levels of CD14/CD163 and SPECT-detected macrophage activity, with CD14 levels particularly associated with radiographic joint space narrowing, osteophyte formation, and clinical pain scores. Interestingly, CD163 as an M2 marker showed specific association with osteophyte progression, suggesting a potential role for M2 macrophages in this aspect of OA pathogenesis. Kraus et al. (25) extended these findings using 99Tc-m-EC20 (Etarfolatide) imaging in human knee OA, detecting activated macrophages in 76% of symptomatic knees with strong correlations to both pain severity and radiographic disease stage. Importantly, FR-β^+^ macrophages were identified in multiple OA-affected joints (fingers, shoulder, ankle), with their presence consistently associated with pain symptoms.

### 2.2 Phenotypic features and functional roles of synovial macrophages

The synovium, a specialized vascular connective tissue that lines the articular capsule, plays a pivotal role in maintaining joint homeostasis by enveloping intra-articular structures (34, 35). The pathological accumulation of macrophages within the synovial lining serves as a diagnostic indicator of synovitis (16, 36), while simultaneously contributing to synovial tissue homeostasis (37). In OA, activated synovial macrophages have been implicated in the pathogenesis of intra-synovial inflammation (38). These cells exhibit remarkable plasticity, undergoing polarization into distinct functional phenotypes in response to local cytokine gradients—a process termed M1/M2 polarization (39). Classical activation (M1 phenotype) occurs following exposure to pro-inflammatory mediators including IFN-γ, LPS, and TNF-α, resulting in enhanced secretion of inflammatory cytokines (TNF-α, IL-1, IL-6, IL-12) and enzymes (COX-2), coupled with diminished IL-10 production (37). Conversely, alternative activation (M2 phenotype) is induced by Th2-derived cytokines (IL-4, IL-13), yielding anti-inflammatory macrophages that promote tissue repair. The M2 population can be further classified into functionally distinct subsets (M2a, M2b, M2c), all participating in extracellular matrix remodeling (40). Single-cell RNA sequencing studies have revealed a spectrum of macrophage activation profiles that do not conform strictly to the M1 or M2 paradigm, indicating that this polarization continuum demonstrates the dynamic adaptability of macrophages to microenvironmental cues. Emerging evidence highlights the prognostic significance of the synovial M1/M2 polarization balance in OA progression. Topoluk et al. (41) employed an innovative OA cartilage-synovium coculture system to demonstrate that synovial macrophage depletion significantly reduced the M1/M2 polarization ratio, concurrently decreasing IL-13 and MMP-13 expression while mitigating cartilage degradation. Complementary clinical findings by Liu et al. (42) revealed elevated M1/M2 polarization ratios in both synovial fluid and peripheral blood of knee OA patients, with positive correlations observed between this ratio and radiographic disease severity, suggesting its utility as a potential biomarker for OA staging.

## 3 Role of synovial macrophage polarization in OA pathogenesis

### 3.1 Synovial inflammation and OA progression

Synovial inflammation serves as a critical initiator of OA, with synovial macrophages, particularly the M1 phenotype, acting as central effectors in disease progression. This inflammatory response is marked by persistent low-grade inflammation, excessive MMP activity, and upregulation of pro-inflammatory cytokines (43). Notably, synovitis often precedes overt cartilage degradation, emphasizing its role as an early pathological hallmark (44). Histological features include synovial lining hyperplasia, leukocyte infiltration, and aberrant angiogenesis (6, 45). Among these cellular changes, M1-polarized macrophages emerge as central mediators of synovial inflammation. Upon activation, these macrophages release a cascade of inflammatory cytokines (TNF-α, IL-1β, IL-6) and pain-inducing neuropeptides, while simultaneously exacerbating oxidative stress and inducing mitochondrial dysfunction through enhanced autophagy (46).

#### 3.1.1 Oxidative stress regulation in OA

Under normal physiological conditions, reactive oxygen species (ROS) serve as critical signaling molecules involved in chondrocyte apoptosis regulation, gene expression modulation, and extracellular matrix (ECM) homeostasis maintenance (47, 48). However, pathological oxidative stress occurs when ROS generation surpasses the antioxidant defense capacity, resulting in structural damage to cellular components and articular tissues (49). Mitochondrial dysfunction is a key contributor to excessive ROS accumulation, which further disrupts intracellular signaling cascades and amplifies inflammatory responses (50). Elevated ROS levels exhibit a strong correlation with M1 macrophage infiltration in OA synovium (51). These classically activated macrophages secrete pro-inflammatory mediators, including IL-1β, IL-6, IL-12, TNF-α, ROS, and inducible nitric oxide synthase (iNOS), which collectively exacerbate synovial inflammation (17, 52, 53). The resulting oxidative stress, driven by ROS and nitric oxide (NO), accelerates chondrocyte apoptosis and cartilage matrix degradation (47). Mechanistically, excessive ROS inhibit the protective PI3K/Akt pathway while activating pro-inflammatory MEK/ERK signaling, further amplifying the inflammatory cascade (47, 54). Zheng et al. (55) demonstrated that populnin alleviates oxidative stress in OA synovium by suppressing NF-κB activation, reducing IL-1β-induced NO production, and downregulating iNOS expression. Macrophage polarization significantly influences cellular redox balance through metabolic reprogramming. M1 macrophages predominantly utilize glycolysis and the pentose phosphate pathway, enhancing ROS and NO generation and perpetuating inflammation. In contrast, M2 macrophages exhibit increased oxidative phosphorylation and express arginase-1 (Arg1), contributing to ROS scavenging and tissue repair (56). Notably, ROS also function as intracellular signaling molecules that reinforce M1 polarization by activating NF-κB and MAPK pathways, creating a self-sustaining inflammatory loop in OA synovitis (57–59).

#### 3.1.2 Mitophagy regulation in OA

Mitochondria serve as the primary organelles for oxidative phosphorylation in eukaryotic cells, playing a pivotal role in cellular energy metabolism (60). Mitophagy, the selective autophagy of damaged mitochondria, is a critical mechanism for maintaining mitochondrial quality control and cellular homeostasis. However, dysregulation of this process can lead to pathological outcomes, as excessive mitophagy may disrupt intracellular balance and trigger apoptotic or necrotic cell death (61). Recent studies have elucidated the relationship between mitophagy and macrophage polarization. Recent studies demonstrated that LPS-stimulated macrophages polarized toward the pro-inflammatory M1 phenotype exhibit elevated intracellular ROS levels, accompanied by mitochondrial dysfunction and marked upregulation of mitophagy-related proteins, including Beclin-1 and PINK1. This enhanced mitophagy activity contributes to the amplification of inflammatory responses. Notably, intervention with 2-deoxy-D-glucose was found to attenuate ROS generation during M1 polarization, thereby reducing the fusion of impaired mitochondria with lysosomes and subsequently suppressing excessive mitophagy, which may offer a potential strategy for mitigating inflammation (62, 63). Conversely, emerging evidence suggests that mitophagy modulation can reciprocally influence macrophage polarization states. For instance, stabilization of mitochondrial membrane potential and pharmacological inhibition of mitophagy have been shown to promote the anti-inflammatory M2 phenotype, thereby alleviating synovial inflammation (64).

### 3.2 Effects on cartilage

Cartilage, an avascular and aneural hyaline tissue, forms the articular surface of bones and is essential for smooth joint movement (65). Mechanical stress-induced cartilage injury triggers chondrocytes and macrophages to release MMPs, which degrade cartilage ECM and exacerbate OA progression (66). The pathological mechanisms of OA involve the imbalance between ECM synthesis and degradation due to elevated catabolic enzyme activity, and chondrocyte apoptosis and cellular senescence. Notably, modulating macrophage polarization, by enhancing the M2 anti-inflammatory phenotype or suppressing the M1 pro-inflammatory response, can promote cartilage repair, restore tissue homeostasis, and mitigate OA progression (67, 68).

#### 3.2.1 Impact on chondrocyte anabolic and catabolic processes

Macrophage-chondrocyte crosstalk via paracrine signaling plays a pivotal role in OA pathogenesis. Activated synovial macrophages release pro-inflammatory cytokines, including IL-1β, TNF-α, and IL-6, which disrupt the chondrocyte microenvironment. These cytokines upregulate MMPs, leading to the breakdown of key ECM components such as type II collagen and aggrecan (69). Importantly, ECM degradation products act as damage-associated molecular patterns (DAMPs), further activating macrophages and perpetuating a self-sustaining cycle of synovial inflammation and cartilage destruction. Experimental evidence highlights the detrimental effects of M1 macrophages on chondrocyte function. Samavedi et al. (70) utilized a 3D co-culture model to demonstrate that chondrocytes exposed to LPS-polarized M1 macrophages exhibited significantly increased secretion of MMPs and inflammatory mediators (IL-1β, TNF-α, IL-6, IL-8, IFN-γ). Furthermore, Zhang et al. (26) identified a novel mechanism wherein M1 macrophage polarization exacerbates OA through chondrocyte-derived R-spondin 2 (Rspo2), which activates β-catenin signaling and amplifies cartilage catabolism.

#### 3.2.2 Impact on chondrocyte apoptosis

**Figure 1:**
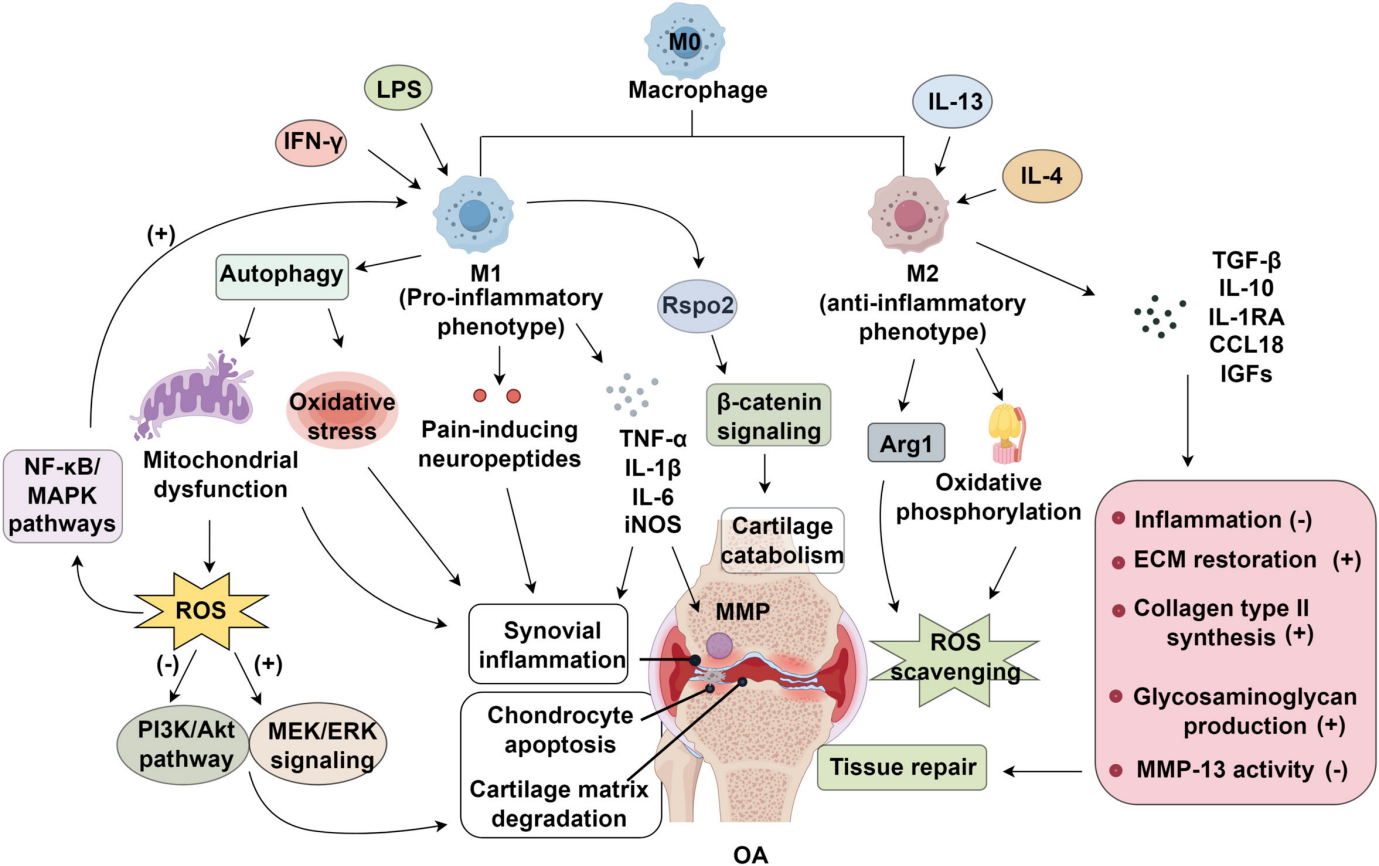
Mechanisms of synovial macrophage polarization in osteoarthritis (OA). Pro-inflammatory M1 macrophages induced by IFN-γ and LPS promote oxidative stress, ROS accumulation, and NF-κB/MAPK activation, driving synovial inflammation and cartilage catabolism. In contrast, IL-4/IL-13–induced M2 macrophages enhance ROS scavenging, ECM restoration, and cartilage repair through Arg1 and β-catenin signaling, thereby suppressing inflammation and promoting tissue regeneration. LPS; IGFs, insulin-like growth factors; ROS, reactive oxygen species; MAPK, mitogen-activated protein kinase; MMP, matrix metalloproteinases; OA, osteoarthritis; MEK, methyl Ethyl Ketone; ERK, Extracellular signal-regulated kinase; PI3K, phosphoinositide 3-kinase; AKT, also known as protein kinase B (PKB); ECM, extracellular matrix.

Growing research highlights the critical role of chronic low-grade synovial inflammation in cartilage degradation, where synovial macrophage polarization serves as a key mediator in OA progression (71). Pro-inflammatory M1 macrophages exacerbate OA pathology by promoting chondrocyte apoptosis, inducing cellular hypertrophy, and facilitating ECM degradation, ultimately leading to cartilage destruction (72). Experimental evidence suggests that targeted depletion of synovial macrophages reduces the M1/M2 ratio, suppresses IL-1 and MMP-13 expression, and mitigates cartilage damage (41). Mechanistically, M1-polarized macrophages in OA synovial tissue release pro-inflammatory mediators, including TNF-α and IL-1β, alongside MMPs, which collectively accelerate ECM breakdown and drive chondrocyte apoptosis (6). Among these cytokines, IL-1β plays a central role in chondrocyte apoptosis by activating the NF-κB and MAPK signaling cascades. Key components of these pathways—such as p50, p52, Rel proteins (Rel, Rel A, Rel B), p38, JNK, and ERK—regulate critical cellular processes, including chondrocyte proliferation, differentiation, and programmed cell death (73, 74). Pharmacological interventions targeting these pathways, such as paeoniflorin, have demonstrated therapeutic potential by modulating the Bax/Bcl-2/Caspase-3 axis and attenuating IL-1β-induced chondrocyte apoptosis (75). In contrast, M2 macrophages exhibit chondroprotective effects by promoting an anti-inflammatory microenvironment and facilitating cartilage repair. Dai et al. (76) demonstrated that type II collagen stimulates M2 polarization, which in turn suppresses chondrocyte apoptosis and hypertrophy while supporting the regeneration of damaged cartilage. This reparative function is partly mediated by TGF-β, a potent anti-inflammatory cytokine that enhances chondrogenic differentiation of bone marrow-derived mesenchymal stem cells (BMSCs) (67). Additionally, M2 macrophages secrete IL-10, IL-1RA, and CCL18, which counteract inflammation, alongside pro-chondrogenic factors such as insulin-like growth factors (IGFs) (77, 78). These macrophages also contribute to ECM restoration by upregulating collagen type II synthesis, enhancing glycosaminoglycan production, and inhibiting MMP-13 activity (79). Beyond direct paracrine effects, macrophage polarization influences chondrocyte fate through modulation of key signaling pathways, including TGF-β, JNK, Akt, NF-κB, and β-catenin. These interactions create a feedback loop wherein ECM degradation further skews macrophage polarization toward a pro-inflammatory phenotype, perpetuating a cycle of inflammation and cartilage deterioration (80) (**Figure 1**).

### 3.3 Impact on chondrogenic differentiation of mesenchymal stem cells

Mesenchymal stem cells (MSCs), as multipotent stromal cells, are widely distributed in diverse tissues such as bone marrow, skeletal muscle, periosteum, and trabecular bone (81, 82). Notably, BMSCs exhibit significant immunomodulatory and anti-inflammatory properties, which contribute to immune suppression and tolerance induction through the downregulation of pro-inflammatory responses. Additionally, BMSCs demonstrate considerable regenerative potential in cartilage repair (83). In the context of OA, BMSCs are recruited to injured joint tissues, where they secrete an array of bioactive molecules, including chemokines, cytokines, and growth factors. These factors play a crucial role in promoting M2 macrophage polarization, thereby facilitating tissue regeneration (67). Furthermore, MSCs exert a dual regulatory effect on macrophage polarization: they suppress the activation of pro-inflammatory M1 macrophages while enhancing the transition to anti-inflammatory M2 phenotypes. Conversely, M1 macrophages negatively influence MSC functionality by impairing their proliferation and viability, exacerbating inflammatory responses, and accelerating cartilage matrix degradation (83). Supporting this, Fahy et al. demonstrated that M1 macrophages significantly inhibit the chondrogenic differentiation capacity of MSCs (84) (**Table 1**).

**Table 1:** Preclinical strategies targeting synovial macrophage polarization in OA and associated outcomes.

Strategy	Mechanisms	Agent(s)	Benefits	Limitations	References
Macrophage depletion	Elimination of synovial macrophages to reduce inflammation	Clodronate liposomes; CD14 ^+^ targeting	Suppresses osteophyte formation; reduces MMPs and synovitis	Lacks subtype specificity; may impair reparative functions	(41, 68, 70)
mTOR/SIRT1 modulation	Alters macrophage polarization via metabolic regulation	Rapamycin; SIRT1 agonists	Reduces M1/M2 ratio; downregulates IL-1β and MMP-13; delays OA	Systemic effects; limited joint-specific delivery	(26, 85)
Type II collagen–induced M2	Induces anti-inflammatory phenotype; promotes regeneration	Squid-derived type II collagen	Enhances TGF-β/IGF; protects chondrocytes; supports repair	Heterogeneous efficacy; limited monotherapy potential	(69, 76)
Anti-inflammatory phytochemicals	Inhibits M1 signaling (NF-κB, MAPK)	Magnolol; Paeoniflorin	Downregulates inflammation; protects cartilage	Off-target effects; long-term immunosuppression risk	(86–90)
Coculture macrophage depletion	Reduces M1/M2 ratio and inflammatory mediators	Clodronate	Decreases IL-1β and MMP-13; mitigates ECM degradation	Ex vivo model; lacks *in vivo* validation	(39, 41, 72, 80)
MSC-mediated modulation	Promotes M2 phenotype via paracrine immunosuppression	BMSC-secreted factors	Enhances regeneration; suppresses M1-driven inflammation	M1-mediated suppression of MSCs; donor variability	(81–84)

## 4 Targeting macrophages as a therapeutic strategy for osteoarthritis

Synovial macrophage depletion has elucidated their pivotal role in OA pathogenesis. Clodronate-loaded liposomes were first shown to effectively ablate synovial macrophages in mice, reducing TGF-β–mediated osteophyte formation (39). This effect was consistently observed across diverse OA models (DMM, CIAOA), reinforcing macrophage-driven osteophyte development (91–93). Subsequent studies also reported decreased VDIPEN levels and MMP-3/MMP-9 expression following depletion, suggesting direct involvement in cartilage matrix degradation (94). However, the lack of macrophage subtype specificity in such approaches limits their translational potential. To improve therapeutic precision, manipulation of mTOR signaling was employed to skew macrophage polarization, demonstrating M1-driven OA exacerbation versus M2-mediated protection (26). Similarly, clodronate treatment in an ex vivo OA model led to a reduced M1/M2 ratio and attenuated IL-1β, MMP-3 expression, and cartilage degradation (95, 96).

Further studies have explored selective macrophage depletion strategies. Targeting CD14 ^+^ macrophages in OA synovium was shown to reduce IL-1 and TNF-α levels (68), whereas CD14^−^ macrophages exhibited higher TNF-α and IL-1β mRNA expression (97). *In vitro* co-culture experiments revealed that M1 macrophages upregulated MMPs, ADAMTS-5, and pro-inflammatory cytokines, contributing to cartilage breakdown (70). Conditioned media from M1-polarized macrophages elevated IL-1β, IL-6, MMP-13, and ADAMTS-5 while suppressing aggrecan and collagen II synthesis (98). Notably, M2 macrophages failed to counteract M1-mediated cartilage destruction, suggesting that simply promoting M2 polarization may not suffice to reverse OA progression. In contrast, type II collagen derived from squid was found to promote M2 macrophage polarization, enhance TGF-β and IGF expression, inhibit chondrocyte apoptosis, and facilitate cartilage repair (76). Additionally, magnolol was shown to reduce the synovial M1/M2 ratio and alleviate inflammation in OA mice by inhibiting the p38/ERK, p38/MAPK, and p65/NF-κB signaling pathways (86). Supporting this approach, SIRT1 activators administered either intraperitoneally or intra-articularly effectively reduced synovial inflammation, lowered the M1/M2 ratio, and delayed OA progression in murine models (85).

Collectively, these findings underscore the protective role of M2 macrophages and suggest that modulating macrophage polarization may represent a viable therapeutic strategy for OA (17, 87, 99). Despite encouraging preclinical results, current macrophage-targeted therapies face several limitations that hinder clinical translation. First, non-specific depletion or systemic modulation of macrophages may lead to unintended off-target effects, including disruption of physiological macrophage functions essential for tissue repair and host defense. Second, strategies that broadly promote M2 polarization or inhibit M1 phenotypes risk inducing generalized immunosuppression, potentially compromising innate immune responses and increasing susceptibility to infections. Third, variability in patient-specific factors such as baseline immune profiles, metabolic status, and genetic polymorphisms may result in heterogeneous therapeutic responses, posing challenges for personalized treatment optimization. Lastly, the lack of reliable biomarkers to monitor *in vivo* macrophage polarization hinders real-time assessment of therapeutic efficacy (87–90).

## 5 Conclusion

Synovial macrophages play a central role in OA pathogenesis, where their polarization state critically influences inflammatory cascades and tissue homeostasis. Pro-inflammatory M1 macrophages exacerbate synovitis, oxidative stress, and extracellular matrix degradation, whereas M2 macrophages promote resolution of inflammation and tissue repair. Emerging therapeutic approaches, including selective macrophage depletion, mTOR pathway modulation, and SIRT1 activation, have shown efficacy in shifting the M1/M2 equilibrium toward a reparative phenotype, thereby mitigating OA progression. Despite these advances, key challenges persist, particularly in achieving macrophage subtype-specific targeting and bridging the gap between preclinical models and clinical applications. To overcome these challenges, future strategies should emphasize cell-specific delivery systems, such as nanocarrier-mediated or ligand-directed targeting, to selectively reprogram synovial macrophage subtypes within the joint microenvironment. Moreover, combinatory approaches that integrate macrophage modulation with anti-inflammatory agents, regenerative medicine or mechanical joint unloading may yield synergistic benefits. By strategically manipulating macrophage polarization, researchers may unlock innovative treatments capable of not only slowing OA progression but also restoring joint integrity and function.
